# High-energy X-ray micro-lamino­graphy to visualize microstructures in dense planar objects

**DOI:** 10.1107/S1600577522012176

**Published:** 2023-02-03

**Authors:** Masato Hoshino, Kentaro Uesugi, Takuya Imai

**Affiliations:** a Japan Synchrotron Radiation Research Institute, 1-1-1 Kouto, Sayo, Hyogo 679-5198, Japan; b Fukui Prefectural University, 4-1-1 Matsuoka-kenjojima, Eiheiji, Fukui 910-1195, Japan; Australian Synchrotron, Australia

**Keywords:** high-energy X-ray, micro-lamino­graphy, fossils, X-ray refraction contrast

## Abstract

High-energy X-ray micro-lamino­graphy has been developed to observe inner- and near-surface structures in dense planar objects that are not suitable for observation by X-ray micro-tomography. A multilayer-monochromator-based high-intensity X-ray beam with energy of 110 keV was used for high-energy and high-resolution lamino­graphic observations. As a demonstration of high-energy X-ray micro-lamino­graphy for observing dense planar objects, a compressed fossil cockroach on a planar matrix surface was analyzed with effective pixel sizes of 12.4 µm and 4.22 µm for wide field of view and high-resolution observations, respectively. In this analysis, the near-surface structure was clearly observed without undesired X-ray refraction-based artifacts from outside of the region of interest, a problem typical in tomographic observations. Another demonstration visualized fossil inclusions in a planar matrix. Micro-scale features of a gastropod shell and micro-fossil inclusions in the surrounding matrix were clearly visualized. When observing local structures in the dense planar object with X-ray micro-lamino­graphy, the penetrating path length in the surrounding matrix can be shortened. This is a significant advantage of X-ray micro-lamino­graphy where desired signals generated at the region of interest including optimal X-ray refraction effectively contribute to image formation without being disturbed by undesired interactions in the thick and dense surrounding matrix. Therefore, X-ray micro-lamino­graphy allows recognition of the local fine structures and slight difference in the image contrast of planar objects undetectable in a tomographic observation.

## Abstract 2

The development of synchrotron-radiation-based high-energy X-ray micro-lamino­graphy to visualize microstructures in dense planar objects such as planar fossils is described.

## 1. Introduction

X-ray micro-lamino­graphy (micro-LG) utilizing a synchrotron radiation X-ray source as well as a laboratory source has been employed as a non-destructive three-dimensional (3D) imaging technique for planar objects as a substitute for conventional X-ray micro-tomography (micro-CT) (Helfen *et al.*, 2005 ▸, 2012 ▸, 2013 ▸; Fisher *et al.*, 2019 ▸; Wood *et al.*, 2019 ▸). In the tomographic observation of a planar object, its planar shape essentially hinders visualization of the inner structure due to poor X-ray transmission along the longitudinal direction and a geometrical restriction caused by the relation between the effective field of view (FOV) in the projection image and the sample size in the longitudinal direction. In comparison, X-ray micro-LG enables us to observe the region of interest even for a large-area sample by simply tilting the rotational axis of the sample, which is generally set perpendicular to the planar surface. In previous studies, X-ray micro-LG has been applied to observations of crack initiation and propagation (Moffat *et al.*, 2010 ▸; Xu *et al.*, 2010 ▸; Tsuritani *et al.*, 2015 ▸). In addition, some studies involving cultural heritage and thin biological samples have been successfully conducted with X-ray micro-LG (Krug *et al.*, 2008 ▸; Verboven *et al.*, 2015 ▸). Phase-contrast X-ray micro-LG based on a grating interferometer has also been developed to apply to polymer-based samples (Helfen *et al.*, 2009 ▸; Harasse *et al.*, 2011 ▸; Altapova *et al.*, 2012 ▸).

In the case of dense and thick planar objects such as flat fossils, X-ray micro-LG can also be a promising tool for assessing non-destructive 3D analysis. Since the biological structures in a planar fossil specimen are often preserved parallel to the planar surface of the rock that contains them, such a specimen can be a good candidate for observation with X-ray micro-LG. Although a few studies have reported the lamino­graphic observation of fossils, the applicability of X-ray micro-LG has not been fully explored with a thorough description of the technical background investigation (Houssaye *et al.*, 2011 ▸; Zuber *et al.*, 2017 ▸). The challenge has been that a dense, flattened macroscopic fossil generally requires high-energy X-rays with extensive spatial resolution for practical observation of its inner structures. So far, micro-fossils and high-density materials that can be trimmed into small pieces have been successfully measured with synchrotron-radiation-based high-resolution X-ray micro-CT using lower-energy X-rays (Donoghue *et al.*, 2006 ▸; Tafforeau *et al.*, 2006 ▸; Yin *et al.*, 2015 ▸). However, micro-structures in a macro-scale fossil might have a significant impact on understanding the paleobiology of extinct organisms. From this perspective, the development of a high-resolution X-ray imaging technique that potentially serves non-destructive 3D analyses for such planar macroscopic fossils has been desired.

In the case of high-resolution measurements in the high-energy region, the performance greatly depends on the X-ray photon flux density. High-intensity and high-energy X-rays obtained from large-sized synchrotron radiation facilities are basically suitable for such analyses. In addition, X-ray micro-imaging systems using synchrotron radiation are superior compared with laboratory-based systems in terms of spatial coherence even in the high-energy region. When fossil samples are observed with high-energy X-rays, the effective enhancement of the image contrast is crucial to differentiate fossil inner structures from other, non-biological, micro-scale materials. In this regard, a high degree of the spatial coherence in synchrotron radiation is useful to support high-contrast observations using proper edge-enhancement caused by X-ray refraction (Wilkins *et al.*, 1996 ▸; Yagi *et al.*, 1999 ▸).

In this study, synchrotron-radiation-based X-ray micro-LG employing X-rays higher than 100 keV has been developed for the purpose of observing dense planar objects. To demonstrate its applicability to planar objects such as planar fossils, the image quality obtained from X-ray micro-LG was compared with that obtained from a conventional tomographic geometry to address the validity of the lamino­graphic observation for planar fossil specimens in terms of the image contrast and the spatial resolution.

## 2. Development of high-energy X-ray micro-LG

### 2.1. Measurement setup

High-energy X-ray micro-LG was developed at the medium-length bending-magnet beamline BL20B2 at SPring-8 (Goto *et al.*, 2001 ▸). A double-multilayer monochromator (DMM) optimized for an output energy of 110 keV was used to utilize high-energy X-rays with higher photon flux density. The energy resolution (Δ*E*/*E*) was 0.9%. Lower-energy components from total reflection were reduced by a 0.3 mm-thick copper filter. Details of the DMM are given elsewhere (Koyama *et al.*, 2023 ▸). Downstream experimental hutches at BL20B2 located more than 200 m from the source were used in this study. A long propagation distance from the source to the experimental hutches makes it possible to use an X-ray beam with higher spatial coherence which is more advantageous for observing fossil specimens with X-ray refraction contrast, namely X-ray phase contrast. A goniometer was installed on the sample stage in order to tilt the rotational axis of the sample for X-ray micro-LG. The goniometer could be operated with a tilt angle range from 0°, which is normal to the optical axis of the X-ray beam, to 30° for X-ray micro-LG. Accordingly, a lamino­graphic setup could be quickly switched to a tomographic one for comparison of resulting images of the same sample.

X-ray transmission images were recorded with a lens-coupled visible-light conversion-type X-ray imaging detector (Uesugi *et al.*, 2011 ▸). For a wide FOV measurement up to 50 mm in width, a dedicated X-ray detector for high-energy X-ray micro-imaging developed in our previous study was used (Hoshino *et al.*, 2020 ▸). As a scintillating material, a Lu_3_Al_5_O_12_ (LuAG) transparent ceramics plate of thickness 500 µm was employed (Kameshima *et al.*, 2019 ▸). A visible-light image demagnified by the lens system was detected with a high-definition CMOS camera [C13949-50U; 4096 (H) × 3008 (V) pixels, 3.45 µm pixel^−1^, 12-bit ADC; Hamamatsu Photonics]. The propagation distance from the sample to the detector was set to 8 m to utilize the X-ray refraction contrast effectively. The effective pixel size and the width of the projection image were 12.4 µm and 50.8 mm, respectively. On the other hand, a high-resolution X-ray imaging detector was also prepared to demonstrate the high-resolution observation for micro-scale structures within the fossil specimens. In this high-resolution detector, an AA60 beam-monitor (Hamamatsu Photonics) was used. Additionally, as a scintillating material, a LuAG single-crystal plate of thickness 500 µm was employed. As in the wide FOV measurement, a high-definition CMOS camera was used for high-resolution observations. In these observations, the propagation distance from the sample to the detector was set to 3 m. The effective pixel size was 4.22 µm.

A filtered back-projection (FBP) method considering the tilt angle of the rotational axis was used for reconstructing a sectional image in X-ray micro-LG (Hoshino *et al.*, 2011*a* ▸). A conventional FBP method was used for X-ray micro-CT. In both reconstructions under the FBP method, a Chesler filter was applied to the projection images. In this study, phase retrieval was not employed to directly utilize the edge-enhancement effect by X-ray refraction in the reconstructed images.

### 2.2. Specimens

**Figure 1:**
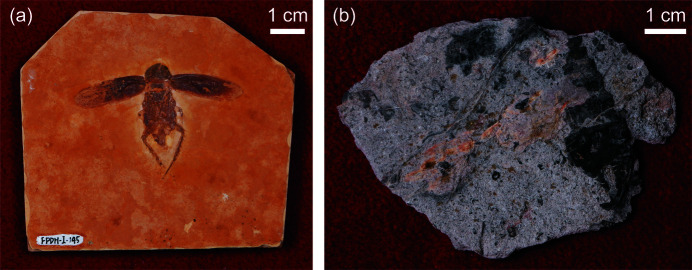
Photographs of the specimens. (*a*) FPDM-I-145 preserved on the planar surface. (*b*) FPDM-I-2337 preserving various invertebrate remains including gastropods.

Specimens employed in this study include FPDM-I-145 and FPDM-I-2337 deposited in Fukui Prefectural Dinosaur Museum (FPDM), Katsuyama, Fukui, central Japan. FPDM-I-145 contains a fossilized cockroach (Blattaria indet.) from the late Aptian Crato Formation, Araipe Basin, Brazil. The Crato Formation is known for its high-fidelity preservation of flattened fossil insects. They are preserved in forms of pyrite and goethite, replacing the original cuticle, and of calcium phosphate, replacing the original internal soft tissues (Barling *et al.*, 2020 ▸), as FPDM-I-145 most probably is. These fossilized insects are preserved within finely laminated limestones that tend to split into a form of plates, revealing insect fossils on its surface. FPDM-I-145 is 78.1 mm long, 68.8 mm wide and 7.9 mm thick, including the fossilized cockroach and matrix preserving it [Fig. 1 ▸(*a*)]. FPDM-I-2337 is platy and contains abundant invertebrate remains from the Middle Permian Akasaka Limestone, Gifu, central Japan. The fossil remains are preserved as dark calcium carbonate and are largely composed of gastropods including *Vebericochilis sp*. FPDM-I-2337 is 81.7 mm long, 57.7 mm wide and 6.9 mm thick at the thickest part (at which the largest individual of *Vebericochilis sp.* is preserved). Numerous macro- and micro-fossils are observed on the matrix surface [Fig. 1 ▸(*b*)].

In X-ray micro-LG, FPDM-I-145 was employed to demonstrate its effectiveness for the observation of a flattened fossil preserved on a planar surface, and FPDM-I-2337 for the observation of characteristic inner structures inside the dense planar object. These samples were held with a commercially available plastic container with elastic membranes (Boxel – Precision Instrument Transport Storage Container, No.32, AS ONE, Japan) which supported the specimens even under the tilted condition in the lamino­graphic observation. In the following measurements, the rotational axis was set to be perpendicular to the planar surface of each specimen.

## 3. Results

**Figure 2:**
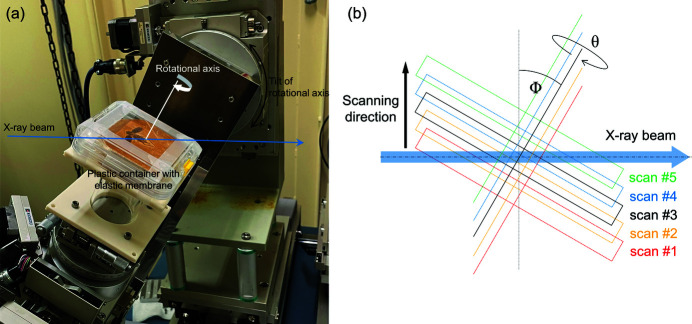
(*a*) Measurement setup for X-ray micro-lamino­graphy of planar specimens. In this photograph, the tilt angle of the rotational axis was set to 30° from the normal direction to the X-ray beam. (*b*) Schematic drawing of the wide FOV observation with a vertical sample scan.

**Figure 3:**
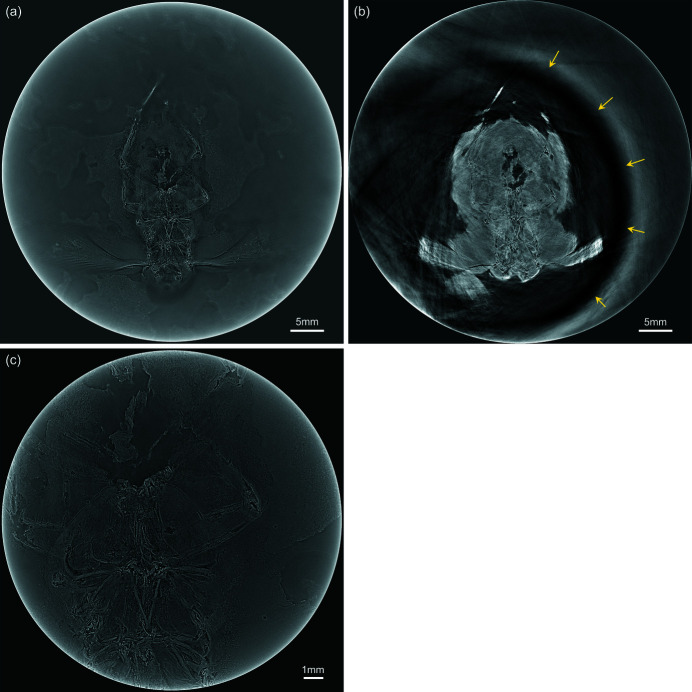
Sectional images of Blattaria indet. (FPDM-I-145) analyzed by (*a*) X-ray micro-LG and (*b*) X-ray micro-CT, in which the same sectional planes are presented. The arrows indicate part of the elastic membrane that hold the specimen. (*c*) High-resolution sectional image of Blattaria indet. (FPDM-I-145) observed with an effective pixel size of 4.22 µm.

Fig. 2 ▸(*a*) shows the experimental arrangement of FPDM-I-145 on the sample stage. The measurement conditions in the wide FOV observation for FPDM-I-145 with X-ray micro-LG were as follows: 7200 projections from 360° rotation, 150 ms exposure time and 30° rotational axis tilt angle. In a lamino­graphic measurement, an effective vertical field of view (v_FOV) of more than h_FOVsinΦ is basically required in the projection images to reconstruct a full-field sectional image, where h_FOV is the horizontal field of view that is equal to the width of the sectional image, and Φ is the tilt angle of the rotational axis. Since h_FOV was 50.8 mm in the wide FOV observation, v_FOV should be larger than 25.4 mm for a 30° tilt angle. However, the effective vertical width of the high-energy X-ray beam at the sample position was limited to approximately 10 mm. To overcome this limitation, the sample was scanned along the vertical direction step by step at every 360° rotation as shown in Fig. 2 ▸(*b*). FPDM-I-145 was scanned five times with a stepping amount of 8 mm per step. Then, five-part sub-projection images were combined before reconstruction to make a single projection image with v_FOV of 40 mm. A sectional image of FPDM-I-145 parallel to the planar surface is shown in Fig. 3 ▸(*a*). For comparison, the same specimen was analyzed under tomographic conditions (Φ = 0) with the only difference being the tilt of the rotational axis. The following measurement conditions for X-ray micro-CT were applied: number of projections 3600 from 180° rotation and exposure time 150 ms. A sectional image obtained from X-ray micro-CT at the same sectional plane is shown in Fig. 3 ▸(*b*). In the tomographic image, part of the elastic membrane that would have been located near the sectional plane could be seen as indicated by arrows. In addition, some artifacts from undesired interactions with the structures near the surface significantly affect the image quality (see the video in the supporting information). The penetration path length of the X-ray beam in the sample tends to be longer under tomographic conditions so the rotational axis is set to be normal to the planar surface. Therefore, the required signals from the region of interest can be disturbed by any material in the beam path outside of it, causing a noisy image due to a decrease in transmission and overlapping of streak-like artifacts. Consequently, the image quality defined by the spatial resolution and the image contrast is degraded. In comparison, micro-scale morphologies of FPDM-I-145 are clearly observed in X-ray micro-LG. The aid of the high-resolution detector reveals the veins in the wing membranes and ventral side of the abdomen covered by the matrix. Additionally, we conducted high-resolution X-ray micro-LG with the following experimental conditions: number of projections 7200 from 360° rotation, exposure time 200 ms and tilt angle 30°. A high-resolution sectional image around the abdomen is shown in Fig. 3 ▸(*c*). Micro-structures of the abdomen in the ventral side, which X-ray micro-CT failed to capture, is clearly visualized. From this point of view, localized high-resolution observations and multi-scale observations even in a large-scale sample are outstanding features of X-ray micro-LG.

**Figure 4:**
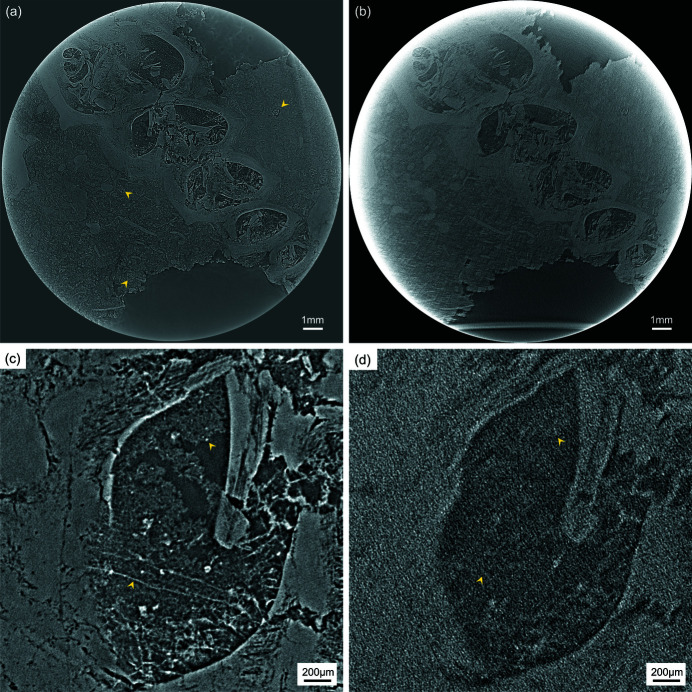
Sectional images of a *Vebericochilis* shell in FPDM-I-2337 analyzed with (*a*) X-ray micro-LG and (*b*) X-ray micro-CT. The arrows in (*a*) indicate small fossil remains in the surrounding matrix. Magnified images at the upper middle region in sectional images obtained from (*c*) X-ray micro-LG and (*d*) X-ray micro-CT. The arrows in (*c*) and (*d*) indicate filamentous structures and the matrix infilling the shell.

To demonstrate the observation of fossils within a dense planar matrix, FPDM-I-2337 was analyzed with the high-resolution detector. The measurement conditions in high-resolution X-ray micro-LG were as follows: number of projections 7200 from 360° rotation, exposure time 200 ms and tilt angle 30°. A sectional image of a *Vebericochilis* shell is shown in Fig. 4 ▸(*a*). Micro-scale morphology in the shell and small fossil remains in the surrounding matrix indicated by arrows in Fig. 4 ▸(*a*) are clearly visualized. The small remains in the matrix likely represent fragmentary invertebrate shells and micro-fossils including foraminifera typical of Akasaka Limestone. For comparison, the same region of interest was observed with X-ray micro-CT; the measurement conditions were as follows: number of projections 3600 from 180° rotation and exposure time 200 ms. A sectional image obtained from X-ray micro-CT is shown in Fig. 4 ▸(*b*). The image quality is degraded by lower X-ray transmission and undesired interactions similar to the images obtained from FPDM-I-145. The magnified images shown in Figs. 4 ▸(*c*) and 4 ▸(*d*) indicate the difference in the image quality in more detail. For example, filamentous structures as well as the matrix infilling the shell indicated by arrows are distinguishable only in X-ray micro-LG.

Notably, the angular increments in both tomographic and lamino­graphic measurements were equal at 0.05° per projection. Nevertheless, significant improvement of the image quality resulted by simply tilting the rotational axis.

## 4. Discussions

In this study, the validity and superiority of high-energy X-ray micro-LG for observing planar fossils are successfully demonstrated by comparing the image quality in sectional images obtained from X-ray micro-LG and micro-CT. As shown in Figs. 3 ▸(*b*) and 4 ▸(*b*), a long penetration path length in the matrix surrounding the region of interest causes significant degradation of the image quality due to undesired inter­actions. A major factor degrading the image quality appears to be a decrease in the effective signals due to insufficient transmission along the surrounding matrix. In this regard, X-ray micro-LG was intrinsically valid for such a measurement because the penetration path length in the surrounding matrix is greatly shortened. In addition, the tilted layout in X-ray micro-LG is capable of effectively using X-ray refraction generated around the region of interest to enhance the image contrast without disturbance from the surrounding matrix. On the other hand, streak-like artifacts that might be caused by undesired X-ray refraction from the planar surface in the tomographic condition degrade the quality of the sectional images near the surface as shown in Fig. 3 ▸(*b*) (see also the video in the supporting information). As for the streak-like artifacts, it is well known that they occur along the back-projection angle in the tomographic reconstruction when the projection image contains areas with significantly different absorption coefficients. In this case, only a sectional plane that includes such areas is affected. In comparison, refracted X-rays from the planar surface near the sectional plane under reconstruction may also affect the resultant image even though they do not originate from the structure in the plane. This is a result of the strong edge-enhancement caused by X-ray refraction spreading over several pixels in the projection image when the X-ray beam path is parallel to the surface. When a planar object is measured under X-ray micro-CT using X-rays with high degrees of spatial coherence, especially in the case where a region of interest is found near the surface, the influence of such artifacts must always be taken into account even if the observation is made with high-energy X-rays. In comparison, no projection image generally includes the angular region where the incident X-ray beam becomes parallel to the planar surface in X-ray micro-LG. This feature saves the image quality in the sectional image from undesired interactions caused outside the region of interest and enables high-resolution observations focused on the micro-structures of planar objects.

In this study, a commercially available plastic container with elastic membranes was used to hold indefinite-shaped planar specimens. The study demonstrates that the holding performance of the container is adequately applicable to high-resolution observation with an effective pixel size of 4.22 µm, albeit a limitation is the size of the container. While the elastic membrane was vsible in the sectional image of FPDM-I-145 under X-ray micro-CT, as in Fig. 3 ▸(*b*), the X-ray micro-LG observation under the same experimental settings except for a tilt of the rotational axis did not cause such an issue, likely benefitting from a specific effect in the lamino­graphic measurement. In X-ray micro-LG, the elastic membrane covered the whole area of the region of interest in the projection images. Therefore, the membrane was regarded as a transparent and uniform material on the projection images like a thin filter and caused no interaction with the X-ray beam. In addition, all interactions of the X-ray beam and the membrane are generally small in the 110 keV high-energy region compared with in the lower-energy region. Consequently, the membrane located near the sectional plane did not provide any effect on the sectional image as shown in Fig. 3 ▸(*a*). In comparison, the membrane presented in the X-ray micro-CT sectional image was likely caused by strong X-ray refraction generated at the boundary between the membrane and the air in the angular region where the membrane became parallel to the X-ray beam. With respect to more generalized measurements for planar specimens and more irregularly shaped specimens, such as those containing protrusions, a high-performance 3D printer combined with a precision 3D outline scanner might be useful for creating a dedicated sample holder. The female holder precisely adapted to the outline of the indefinite-shaped specimen should enable a higher spatial resolution to be achieved, even under the tilted lamino­graphy layout

In the X-ray micro-LG observation, the artifacts specific to lamino­graphy have been discussed in previous studies (Helfen *et al.*, 2005 ▸, 2006 ▸; Hoshino *et al.*, 2011*b* ▸; Xu *et al.*, 2012 ▸). The boundary structure along the rotational axis, which is normal to the planar surface, becomes unclear due to missing information in the Fourier domain in the lamino­graphy reconstruction. In addition, an inherent artifact also tends to appear at the cross section away from the central sectional plane. In this case, a distinct structure in the central sectional plane tends to appear as isotropic blurred images in other sectional planes along the rotational axis. However, no significant impact was detected to recognize the micro-structures in the current study as shown in Figs. 3 ▸ and 4 ▸. Since typical fossil specimens and their matrices are composed of minerals with similar material features, little change in density or absorption coefficients is present, causing little influence of the blurred images derived from the distinct structure. Thus, the material features in fossil specimens positively affected the quality of the observation with minimal effects from the artifacts specific to X-ray micro-LG, while measurements using synchrotron radiation with high degrees of spatial coherence are necessary for visualization of such similar material features. Moreover, micro-scale morphologies in compressed planar specimens can be observed in the sectional plane normal to the rotational axis in which the influence of artifacts specific to lamino­graphy can be reduced. Therefore, it can be concluded that high-energy X-ray micro-LG is a compatible method for analyzing microscopic morphologies and inner structures of planar fossil specimens and fossils within planar matrices. On the other hand, it would be more effective to use an iterative reconstruction technique to reduce artifacts specific to lamino­graphy (Fisher *et al.*, 2019 ▸). This approach would allow for a wider range of application of high-energy X-ray micro-LG to various materials.

X-ray micro-LG has numerous applications for visualizing the micro-scale morphology of compressed fossil specimens within a planar matrix. As in FPDM-I-145 from the Crato Formation, multiple well-known fossil sites produce animal remains that are preserved flattened, sometimes with internal organs and epidermal structures, on finely laminated rocks that typically break into a planar matrix with such remains exposed on top (*e.g.* Burgess Shale, Bear Gulch Limestone, Holzmaden Shale, Solnhofen Limestone, Yixian Formation, Santana Group and Messel Oil Shale). Under such preservation, physical observation of the unexposed side of the specimen is virtually impossible without removing the matrix completely, which involves serious risk of damaging it. Previously, tomographic analyses on flattened fossils on a planar matrix failed to capture their morphological details due to X-ray artifacts and inadequate X-ray transmission along the direction parallel to the matrix plane. X-ray micro-LG can negate such problems and can be applied to numerous specimens that are flattened onto planar matrices.

## 5. Conclusion

High-energy X-ray micro-LG at an X-ray energy of 110 keV has been developed, and two specimens with planar fossil materials were observed to demonstrate the applicability of the technique to dense planar objects for which X-ray micro-CT fails to capture adequate structural information. While the present study focuses on planar fossil specimens, the high-penetration power of the high-energy X-rays has potential for various applications. As further applications using high-energy X-ray micro-LG, non-destructive testing of electronic devices and *in situ* observation of a fuel cell battery would be potential candidates. Since localized high-resolution observations can be regarded as outstanding features of X-ray micro-LG, upgrading the system so that it is capable of conducting measurements with an effective pixel size of 1 µm or smaller will be an interesting approach to reveal micrometre-scale features for geological, biological and engineering investigations.

## Supplementary Material

Supplementary Figures S1, S2 and S3. DOI: 10.1107/S1600577522012176/tv5043sup1.pdf

Video comparing sequential slices of FPDM- I-145 along the depth direction. DOI: 10.1107/S1600577522012176/tv5043sup2.avi
