# Supplementary material for: High-energy X-ray micro-lamino­graphy to visualize microstructures in dense planar objects
Source: J Synchrotron Radiat. 2023 Feb 3;30(Pt 2):400–6. doi: 10.1107/S1600577522012176 (PMC10000797; doi:10.1107/S1600577522012176)
Supplement: Supplementary file 1 [file s-30-00400-sup1.pdf]

JOURNAL OF SYNCHROTRON RADIATION

Volume 30 (2023)

Supporting information for article:

High-energy X-ray micro-laminography to visualize microstructures in dense planar objects

Masato Hoshino, Kentaro Uesugi and Takuya Imai

S1. High-resolution sectional images obtained from X-ray micro-laminography

Additional sectional images of FPDM-I-145 and FPDM-I-2337 obtained from high-resolution X-ray micro-LG are shown in Fig. S1 and S2, respectively. A magnified image at the square region in Fig. S2 is shown in Fig. S3(a). A line profile measured at the periodic structure indicated by the arrowed line is shown in Fig. S3(b). The periodic structure of approximately 23 μm (5.5 pixels) can be clearly resolved.

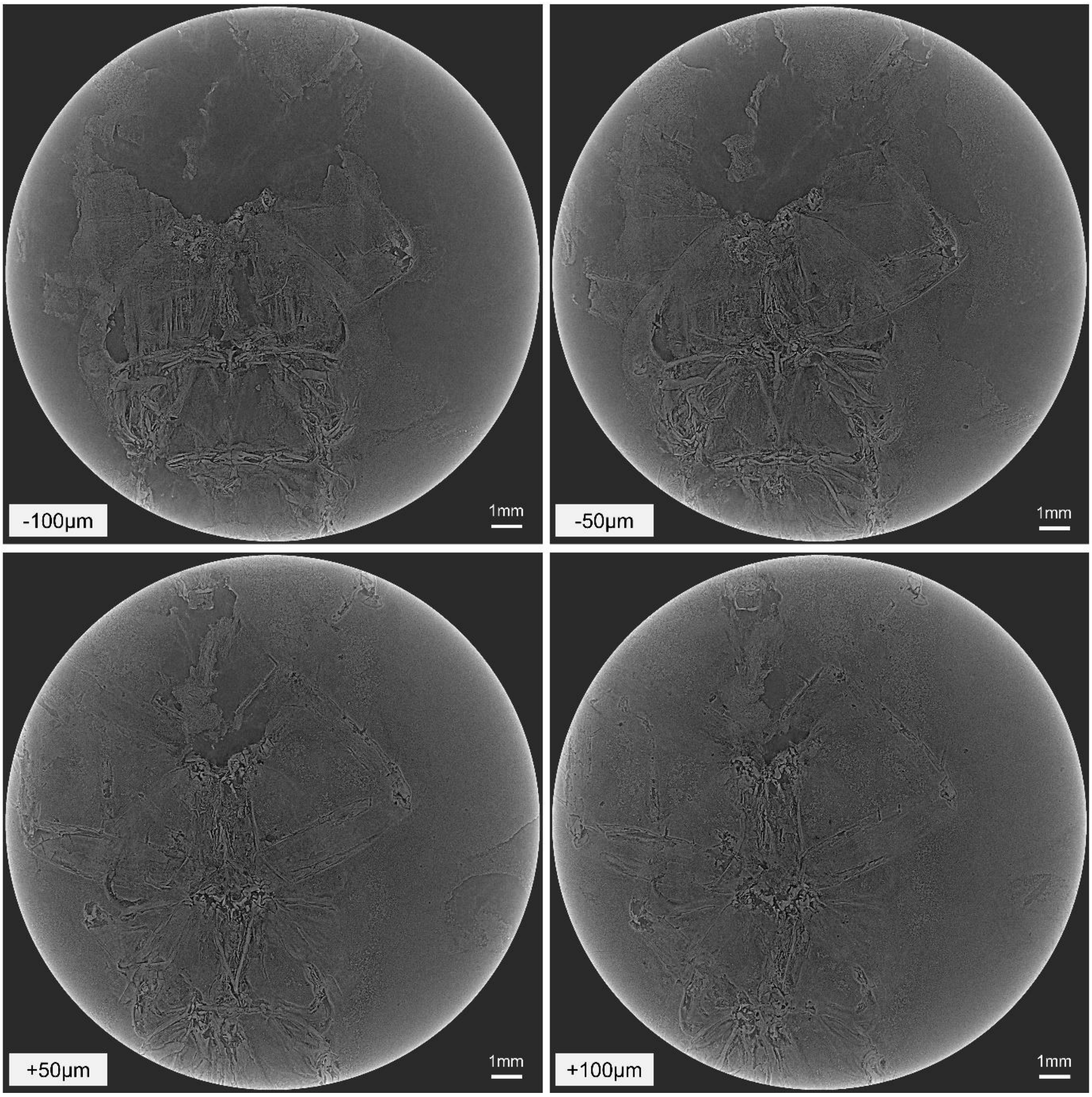

Figure S1 Sectional images of a FPDM-I-145. Numerical value shown in lower left in each image represents a distance from the sectional plane shown in Fig. 3(c).

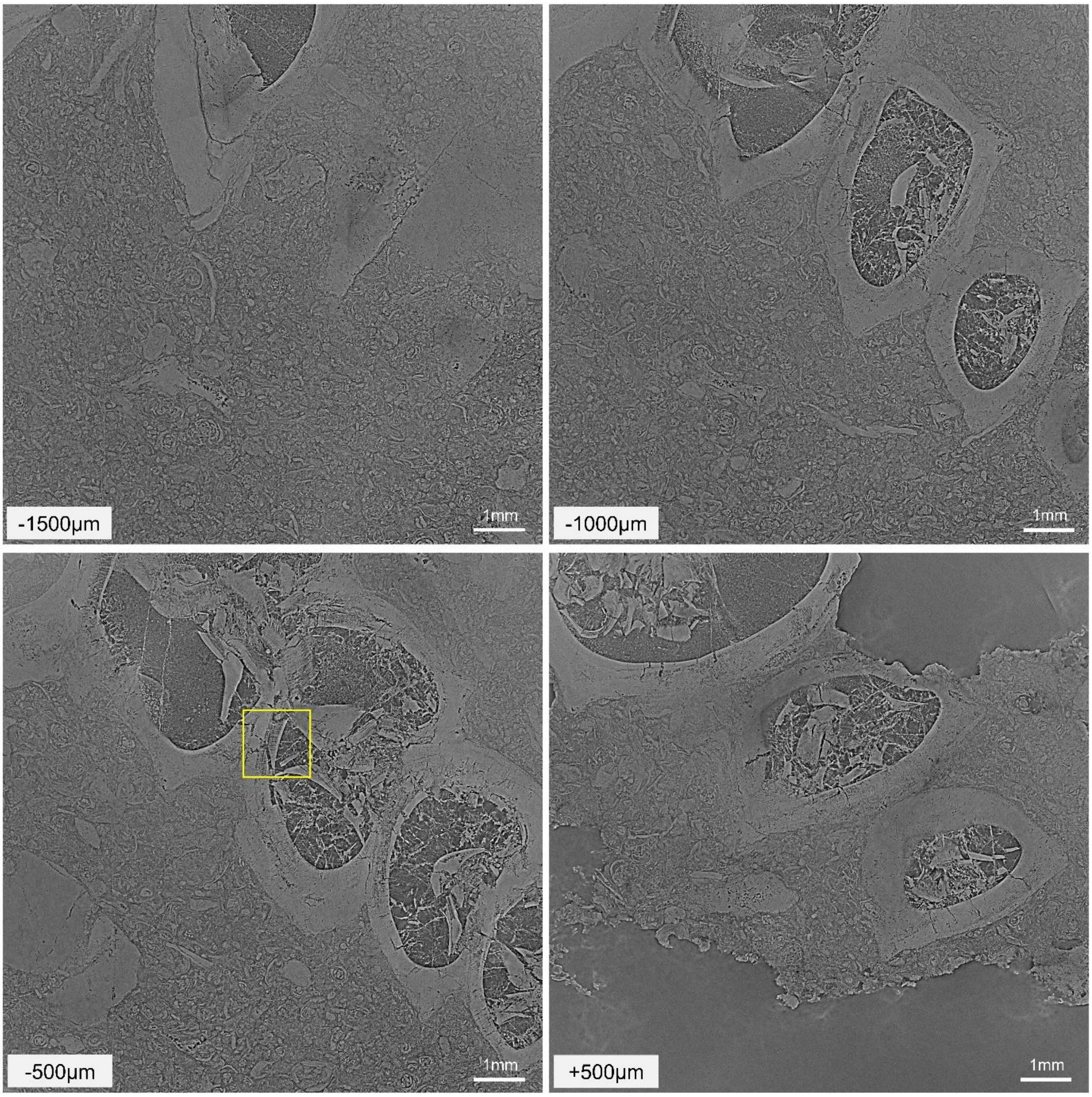

Figure S2 Sectional images of FPDM-I-2337. Numerical value shown in lower left in each image represents a distance from the sectional plane shown in Fig .4(a).

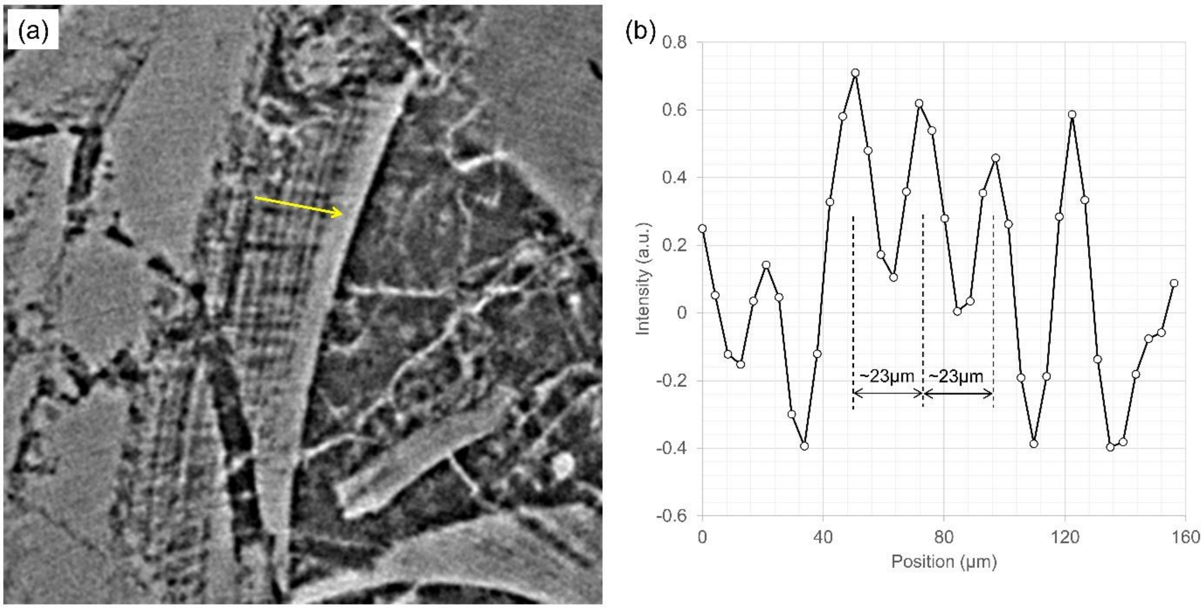

Figure S3 (a) Magnified image at the square region shown in Fig. S2. (b) Line profile measured at the arrowed line in (a).

S2. Sequential slices along the depth direction

A video in .avi format comparing sequential slices of FPDM- I-145 along the same depth direction under X-ray micro-CT (left) and X-ray micro-LG (right).
